# Acute effects of air pollutants on pulmonary function among students: a panel study in an isolated island

**DOI:** 10.1186/s12199-017-0646-3

**Published:** 2017-04-04

**Authors:** Yoshiko Yoda, Hiroshi Takagi, Junko Wakamatsu, Takeshi Ito, Ryouhei Nakatsubo, Yosuke Horie, Takatoshi Hiraki, Masayuki Shima

**Affiliations:** 1 Department of Public Health, Hyogo College of Medicine, 1-1 Mukogawa-cho, Nishinomiya, Hyogo 663-8501 Japan; 2 National Institute of Technology, Yuge College, Kamijima, Ehime Japan; 3 Hyogo Prefectural Institute of Environmental Sciences, Kobe, Hyogo Japan

**Keywords:** Allergy, Asthma, Air pollution, Isolated island, Pulmonary function, Panel study

## Abstract

**Background:**

Many epidemiological studies on the health effects of air pollutants have been carried out in regions with major sources such as factories and automobiles. However, the health effects of air pollutants in regions without major sources remain unclear. This study investigated the acute effects of ambient air pollution on pulmonary function among healthy students in an isolated island without major artificial sources of air pollutants.

**Methods:**

A panel study was conducted of 43 healthy subjects who attended a school in an isolated island in the Seto Inland Sea, Japan. We measured the forced expiratory volume in 1 s (FEV_1_) and peak expiratory flow (PEF) every morning for about 1 month in May 2014. Ambient concentrations of particulate matter ≤ 2.5 μm in diameter (PM_2.5_), particulate matter between 2.5 and 10 μm in diameter (PM_10-2.5_), black carbon (BC), ozone (O_3_), and nitrogen dioxide (NO_2_) were measured. The associations between the concentrations of air pollutants and pulmonary function were analyzed using mixed-effects models.

**Results:**

A decrease in FEV_1_ was significantly associated with BC concentrations (−27.28 mL [95%confidence interval (CI):−54.10,−0.46] for an interquartile range (IQR) increase of 0.23 μg/m^3^). The decrease in PEF was significantly associated with indoor O_3_ concentrations (−8.03 L/min [95% CI:−13.02,−3.03] for an IQR increase of 11 ppb). Among subjects with a history of allergy, an increase in PM_2.5_ concentrations was significantly associated with low FEV_1_. In subjects with a history of asthma, an inverse association between the indoor O_3_ concentration and pulmonary function was observed.

**Conclusions:**

Our results demonstrate that increases in BC and O_3_ concentrations have acute effects on the pulmonary function among students in an isolated island without major artificial sources of air pollutants.

**Electronic supplementary material:**

The online version of this article (doi:10.1186/s12199-017-0646-3) contains supplementary material, which is available to authorized users.

## Background

Many studies have reported the influence of air pollutants on the respiratory system [1–7]. Among air pollutants, particulate matter ≤2.5 μm in diameter (PM_2.5_) induces an inflammatory response of the airway when inhaled, reducing the pulmonary function [8–11]. In addition, it is known that exposure to black carbon (BC), a component of PM_2.5_, impairs the lung function in children [12]. Many studies involving patients with respiratory diseases, such as asthma [13–15] and chronic obstructive pulmonary disease (COPD) [16, 17], have been carried out to evaluate the acute effects of air pollution. In addition, these studies have been primarily conducted in urban areas with a high concentration of air pollutants, involving factories and automobiles [18, 19]. Furthermore, most studies adopted concentrations at monitoring stations in the neighborhood to evaluate the influence of air pollutants on the respiratory system.

However, the results of studies among patients with specific diseases or epidemiological studies in areas with a high concentration of air pollutatnts are not always adaptable for a general population. Few epidemiological studies have evaluated the health effects of changes in the concentration of air pollutants in an environment where healthy persons live. Furthermore, the duration of indoor daily activities is longer than that of outdoor activities; therefore, when concentrations at monitoring stations in the neighborhood were used, exposure assessment may not have been accurately performed.

In this study, we repeatedly performed pulmonary function tests of healthy subjects in an isolated island without major artificial sources of air pollutants, and evaluated the acute effects of changes in the concentrations of air pollutants measured in indoor and outdoor spaces on the respiratory system among the subjects.

## Methods

### Study design and subjects

**Fig. 1:**
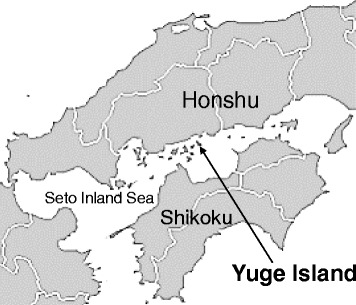
Location of Yuge Island in the Seto Inland Sea of Japan

Yuge Island is an isolated island in the Seto Inland Sea, Japan (Fig. 1). Pulmonary function tests among students were conducted every day between May 12 and June 9, 2014. In addition, the atmospheric environment was measured during the same period. On this island, road traffic is light, but there may be little influence of automobile exhausts. Furthermore, there is no large factory, and there are no artificial sources of air pollutants other than ships; the island may be a clean-air area. However, recently, the influence of transported air pollutants derived from the Asian Continent has been indicated [20]. To investigate the relationship between the atmospheric environment and pulmonary function, we conducted pulmonary function tests among subjects who had stayed in Yuge Island over a long period of time; students attending a school on this island were requested to participate in this study. The subjects were 43 students from whom written informed consent was obtained (aged 15–16 years, mean: 15.1 years, 29 males and 14 females). Prior to this study, its protocol was approved by the Ethics Review Board of Hyogo College of Medicine.

### Assessment of the effects on health

Using a standard questionnaire [21], respiratory symptoms and the presence or absence of a history of asthma or allergy were evaluated. Subjects who selected “Yes” to the question “Have you ever been diagnosed with asthma?” were regarded as having a history of asthma. Those who selected “Yes” to the question “Have you ever been diagnosed with allergic rhinitis?” or “Have you ever been diagnosed with pollinosis?” were regarded as having a history of allergy.

For pulmonary function testing, an electronic peak flow meter (Vitalograph 2110, Vitalograph Ltd., Buckingham, U.K.) was delivered to each subject, and self-measurement of the peak expiratory flow (PEF) and forced expiratory volume in 1 s (FEV_1_) was conducted before the start of the first lesson every morning from Monday until Friday.

### Measurement of the atmospheric environment

To measure the atmospheric environment, an automatic particulate matter-measuring device (SPM-613D, Kimoto Electric Co., Ltd., Osaka, Japan) was installed on the roof of the school, and PM_2.5_, particulate matter between 2.5 and 10μm in diameter (PM_10-2.5_), and BC were measured continuously. In addition, the outdoor concentrations of ozone (O_3_) and nitric oxide (NO_2_) were measured using a passive sampler (Ogawa & Co., Ltd., Kobe, Japan) and a filter badge NO_2_ (Advantec Co. Ltd., Tokyo, Japan), respectively. The sampler and badge were exchanged every 24 h on Monday to Friday for measurement, and 72-h measurement was conducted on weekends. Furthermore, the indoor concentrations of O_3_ and NO_2_ were measured in school classrooms, where the students had stayed for most of each school day, as described for outdoor measurement. The indoor concentration of PM_2.5_ was measured serially using a digital dust indicators (Model LD-5, Sibata Scientific Technology Ltd., Tokyo, Japan) in the classroom.

### Statistical analysis

The relationships between the indoor and outdoor concentrations of PM_2.5_, O_3_, and NO_2_ were expressed as correlation coefficients. Those between the results of a pulmonary function test and the concentrations of air pollutants were evaluated using a mixed effect model with the rate of change per interquartile range (IQR) increase of each pollutant for 24 h before a pulmonary function test after adjusting for the atmospheric temperature, humidity, and subject’s height. Furthermore, the analyses were performed using two-pollutant models to adjust for potential confounding effects of co-pollutants, for outdoor and indoor air pollutants, respectively. A *p*-value of 0.05 was regarded as significant. For analysis, we used SPSS 22 software (IBM Co., Armonk, NY, U.S.A.).

## Results

The background characteristics of the subjects are shown in Table 1. A total of 868 pulmonary function tests were conducted. The means ± standard deviations of PEF and FEV_1_ were 390.1 ± 110.2 L/min and 2.48 ± 0.74 L, respectively. Of the subjects, 7 (16.3%) had a history of asthma, and 19 (44.2%) had a history of allergy without asthma. Seventeen subjects (39.5%) did not have either history. During the study period, no subject developed respiratory symptoms, such as cough, sputum, or wheezing.

**Table 1:** Characteristics of study subjects

	Male (*n* = 29)	Female (*n* = 14)	Total (*n* = 43)
Height, mean (SD) (cm)	166.4 ± 6.8	156.9 ± 5.1	163.3 ± 7.7
PEF (L/min)	420.8 ± 110.2	320.7 ± 73.7	390.1 ± 110.2
FEV_1_ (L)	2.66 ± 0.75	2.10 ± 0.55	2.48 ± 0.74
Measurements of pulmonary function (n)	(594)	(274)	(868)

*SD* standard deviation; *PEF* peak expiratory flow; *FEV*_1_, forced expiratory volume in 1 s

The mean daily indoor/outdoor concentrations of air pollutants during the study period are shown in Table 2. The mean outdoor concentration of PM_2.5_ was 30.9 ± 12.5 μg/m^3^. Those of PM_10-2.5_ and BC were 22.7 ± 15.4 and 0.55 ± 0.22 μg/m^3^, respectively. The maximum outdoor concentration of PM_2.5_ was 61.6 μg/m^3^, which was measured on the day with yellow sand carried from China to Japan. On the same day, the concentration of PM_10-2.5_ was also high (53.8 μg/m^3^). The mean indoor concentration of PM_2.5_ was 17.0 ± 9.6 μg/m^3^.

**Table 2:** The 24-h mean concentrations of outdoor and indoor air pollutants and meteorological parameters during the study period

	n	Mean	SD	Minimum	Median	Maximum	IQR
Outdoor air pollutants							
PM_2.5_ (μg/m^3^)	29	30.9	12.5	13.7	30.6	61.6	18.4
PM_10-2.5_ (μg/m^3^)	29	22.7	15.4	6.0	16.2	54.2	24.2
BC (μg/m^3^)	29	0.55	0.22	0.26	0.49	1.25	0.23
O_3_ (ppb)	21	44.6	10.3	25.7	46.1	58.5	20.8
NO_2_ (ppb)	21	9.2	3.8	4.2	8.6	16.7	6.3
Indoor air pollutants							
PM_2.5_ (μg/m^3^)	29	17.0	9.6	3.7	16.5	39.3	12.6
O_3_ (ppb)	21	15.9	7.5	1.9	16.2	30.0	11.0
NO_2_ (ppb)	21	6.3	3.0	2.2	5.8	15.3	4.2
Meteorological parameters							
Temperature (°C)	29	20.2	2.1	15.8	19.8	23.9	3.6
Relative humidity (%)	29	71.5	11.1	52.0	69.0	92.0	15.5

*SD* standard deviation; *IQR* interquartile range; *PM*_2.5_ particulate matter ≤ 2.5 μm in diameter; *PM*_10-2.5_ particulate matter between 2.5 and 10 μm in diameter; *BC* black carbon; *O*_3_ ozone; *NO*_2_ nitrogen dioxide

**Fig. 2:**
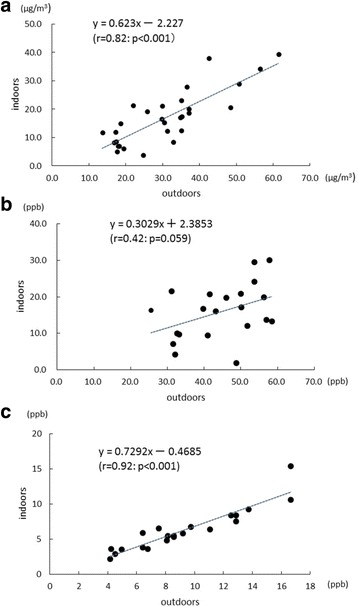
Correlation of indoor and outdoor concentrations of **a** PM_2.5_, **b** O_3_ and **c** NO_2_

There were strong positive correlations between the outdoor and indoor concentrations of PM_2.5_ and NO_2_ (*r* = 0.82 and 0.92, respectively). However, the correlation between those of O_3_ was weak (*r* = 0.42) (Fig. 2). The mean indoor concentrations of PM_2.5_, O_3_, and NO_2_ were lower than the outdoor values.

The associations between the concentrations of air pollutants and changes in parameters of pulmonary function during the study period are shown in Table 3. FEV_1_ decreased significantly in relation to an increase in the BC concentration (−27.28 mL [95% confidence interval (CI):−54.10,−0.46] per IQR increase of 0.23 μg/m^3^). The PEF reduced significantly in relation to an increase in the indoor O_3_ concentration (−8.03 L/min [95% CI:−13.02,−3.03] per IQR increase of 11.0 ppb). There was no significant correlation between the concentrations of the other pollutants and changes in pulmonary function.

**Table 3:** Associations between air pollutants and pulmonary function during the study period

	PEF (L/min)			FEV_1_ (mL)		
	Change^a^	95% CI	*P*-Value	Change^a^	95% CI	*P*-Value
Outdoor air pollutants						
PM_2.5_	−4.57	(−10.67, 1.52)	0.141	−41.19	(−84.38, 1.99)	0.062
PM_10-2.5_	−2.71	(−9.04, 3.62)	0.401	−37.50	(−81.80, 6.79)	0.097
BC	−3.53	(−7.31, 0.25)	0.067	−27.28	(−54.10,−0.46)	0.046
O_3_	−3.84	(−15.42, 7.74)	0.515	−30.93	(−112.84, 50.99)	0.459
NO_2_	2.18	(−3.67, 8.02)	0.466	−28.23	(−69.40, 12.95)	0.179
Indoor air pollutants						
PM_2.5_	−3.21	(−8.63, 2.20)	0.245	−9.58	(−48.50, 29.34)	0.629
O_3_	−8.03	(−13.02,−3.03)	0.002	−20.14	(−55.36, 15.09)	0.262
NO_2_	0.81	(−3.96, 5.59)	0.738	−9.88	(−43.22, 23.46)	0.561

*PEF* peak expiratory flow; *FEV*_1_ forced expiratory volume in 1 s; *CI* confidence interval; *PM*_2.5_ particulate matter ≤ 2.5 μm in diameter; *PM*_10-2.5_ particulate matter between 2.5 and 10 μm in diameter; *BC* black carbon; *O*_3_ ozone; *NO*_2_ nitrogen dioxide

^a^Mean changes in PEF or FEV_1_ associated with an increase in the interquartile range of each air pollutant

We performed analyses using two-pollutant models to adjust for confounding effects of co-pollutants. Additional file 1: Table S1 shows the estimated changes in PEF and FEV_1_ associated with an IQR increase of each pollutant [see Additional file 1]. The FEV_1_ showed a significant decrease in relation to an increase in BC after adjustment for outdoor O_3_. The association between PEF and indoor O_3_ was also significant after adjustment for indoor PM_2.5_ or NO_2_. Furthermore, PEF reduced significantly in relation to an increase in outdoor PM_2.5_ or BC, after adjustment for outdoor NO_2_.

**Fig. 3:**
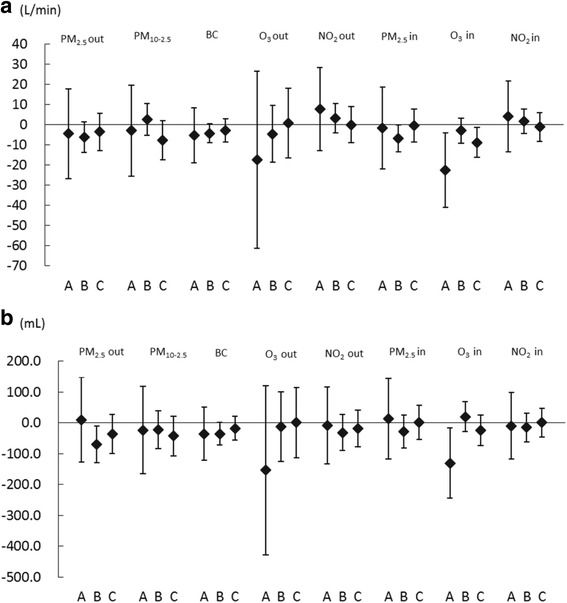
Estimated changes in (**a**) PEF and (**b**) FEV_1_ with increases in air pollutants in relation to a history of asthma or allergy. The estimated changes and 95% confidence intervals for (**a**) PEF and (**b**) FEV_1_ are shown per increase in interquartile range (IQR) of the concentration of air pollutants in relation to a history of asthma or allergy. A: Subjects with a history of asthma. B: Subjects with a history of allergy other than asthma. C: Subjects without a history of asthma or allergy

The associations between the concentrations of air pollutants and PEF with respect to the presence or absence of a history of asthma or allergy are shown in Fig. 3(a). In subjects with a history of allergy, PEF reduced significantly with an increase in the indoor concentration of PM_2.5_ (−6.71 L/min [95% CI:−13.36, −0.06] per IQR increase). In those with a history of asthma, PEF reduced significantly with an increase in the indoor concentration of O_3_ (−22.6 L/min [95% CI:−41.08,−4.13] per IQR increase). On the other hand, the PEF reduced significantly with an increase in the indoor concentration of O_3_ in those without either history (−8.84 L/min [95% CI:−16.28,−1.40] per IQR increase). In those with a history of allergy, FEV_1_ reduced significantly with an increase in the outdoor concentration of PM_2.5_ (−70.0 mL [95% CI:−130.1,−9.94] per IQR increase) (Fig. 3(b)). In addition, FEV_1_ reduced significantly with an increase in the indoor concentration of O_3_ in subjects with a history of asthma (−130.3 mL [95% CI:−243.5,−17.2] per IQR increase).

## Discussion

Even in an isolated island without artificial sources of air pollutants, there were changes in the atmospheric concentrations of air pollutants, indicating that changes in the concentration of BC have an acute influence on the pulmonary function in healthy students. The indoor concentration of O_3_ more markedly influenced pulmonary function compared with its outdoor concentration. In subjects with a history of asthma, pulmonary function reduced significantly with an increase in O_3_ concentration. In those with a history of allergy, it reduced significantly with an increase in PM_2.5_ concentration. These results suggested that subjects with these histories were more susceptible to changes in the concentrations of air pollutants.

Although we thought that this island in the Seto Inland Sea should be a clean-air area, the concentration of PM_2.5_ was considerably high in the island. It was thought that the high concentrations recorded were largely due to transported air pollutants including yellow sand and the emissions from marine ships. Allen et al. showed that PM_2.5_ are transported over long distances and affects distant clean-air areas [22]. It was also reported that transported air pollutants and emissions from marine ships affect the levels of air pollution in Kyushu, Japan [23, 24]. If the emission from marine ships contribute to air quality in the island, the concentration of sulfur dioxide may be also high. However, the concentrations have not been measured around the island.

The results of this study showed that pulmonary function reduced significantly with an increase in the atmospheric concentration of BC, and the association was still significant after adjustment for O_3_. Huang et al. reported that a pulmonary function test in healthy adults after they stayed in a transport hub of a big city for 2 h showed a significant decrease in FEV_1_ related to an increase in BC concentration [25]. Patel et al. conducted a study among school children in New York, and indicated that symptoms, such as wheezing and shortness of breath, deteriorated with an increase in BC concentration [26]. The results of these studies were consistent with those of our study. Diesel emission gas contains a large volume of BC [27]. A study reported that when persons were exposed to diesel emission gas, the bronchial mucosal neutrophil and mast cell counts increased, inducing transient increases in the number of inflammatory cells in the airway and cytokine levels [28]. With an increase in BC concentration, an inflammatory response in the airway may have occurred, thereby reducing pulmonary function. The area investigated in this study was an isolated island with light traffic, which unlike urban areas, and had relatively low concentration of BC. Our results suggested that an increase in BC concentration influences pulmonary function even in such an area with a low air pollutant concentrations. Huang et al. evaluated adults exposed to air pollutants for 2 h in a previous study [25]. In our study, the subjects were adolescent students who attended school for many hours. Because this survey was conducted in an isolated island, the subjects resided both in the island and along the coast. As the 1-month survey was carried out in such a place, the influence of exposure to air pollutants on daily living could be evaluated accurately. Furthermore, most subjects were healthy, and the results of our study may be representative.

The indoor concentration of O_3_ was more markedly associated with pulmonary function compared with its outdoor concentration, and the effect of O_3_ was still significant after adjustment for PM_2.5_ or NO_2_. O_3_ exhibits acute effects on the lungs [29]. When the indoor concentration of O_3_ increased, PEF decreased significantly. However, there was no significant relationship with an increase in the outdoor concentration of O_3_. Altug et al. reported that the PEF among elementary school children decreased significantly with an increase in the concentration of O_3_ [30]. McConnell et al. performed a 5-year follow-up survey of 3535 children without asthma, and indicated that 265 were newly diagnosed with asthma during the follow-up, suggesting that high-intensity exercise in an area with a high concentration of O_3_ promotes the onset of childhood asthma [31]. Furthermore, Amadeo et al. reported that, even at an O_3_ concentration lower than the reference value presented in the WHO guidelines, PEF decreased with an increase in the concentration of O_3_, suggesting the acute effects of O_3_ on the pulmonary function of children [32]. Pulmonary function reduces with an increase in O_3_ concentration, because O_3_ may increase inflammation of the airway as a potent acidic pollutant [10, 33]. Intra-pulmonary O_3_ uptake was reported to peak in the terminal bronchioles [34]. In our study, the relationship with the indoor concentration of O_3_ and pulmonary function was marked. Gaseous substances, such as O_3_, are sometimes generated indoors; therefore, their concentrations in living spaces should be measured and used for exposure assessments.

Neither the indoor nor outdoor concentrations of PM_2.5_ were significantly associated with PEF and FEV_1_ among all the subjects in a single-pollutant model. However, after adjustment for outdoor NO_2_, PEF significantly decreased with an increase in the outdoor concentration of PM_2.5_. Moreover, PEF decreased significantly with an increase in BC after adjustment for NO_2_. These findings may be due to the interactions between NO_2_ and other air pollutants. The results suggested acute effects related to changes in the concentrations of O_3_ and PM_2.5_ in subjects with a history of asthma or allergy. In those with a history of allergy, the PEF and FEV_1_ significantly reduced when the indoor and outdoor concentrations of PM_2.5_ increased, respectively. With respect to the acute effects of PM_2.5_, Huang et al. reported that FEV_1_ decreased by−0.15% (95% CI:−0.28,−0.02%) when the exposure concentration for 2 h increased by 10 μg/m^3^ [25]. Bernals et al. indicated that allergic airway diseases are not merely disorders of the immune system, and that allergic diseases primarily arise from airway epithelium defects resulting from immaturity or caused by environmental insults [35]. In our study, pulmonary function may also have reduced through such a pathophysiological mechanism. Based on the results of this study, there was no relationship between PM_10-2.5_ and changes in pulmonary function. PM_10-2.5_ is deposited in the nasal cavity, pharynx, and upper airway when it is inhaled. However, PM_2.5_ reaches the bronchiolar and alveolar levels in the lungs because its particle diameter is small. A previous study reported that the influence of PM_2.5_ on the respiratory system was more marked than that of PM_10-2.5_ [36]. Based on the results of this study, there was no relationship between the PM_2.5_ concentration and PEF or FEV_1_ in subjects with a history of asthma, but this was possibly because the number of subjects was small.

In subjects with a history of asthma, PEF and FEV_1_ decreased significantly with an increase in the indoor concentration of O_3_. In these subjects, the two parameters decreased slightly with an increase in the outdoor concentration of O_3_. Mortimer et al. reported that PEF in the morning decreased with an increase in O_3_ concentration in children with asthma [37]. In our study, when analyzing the results with respect to medical histories, PEF was associated with an increase in O_3_ concentration in subjects other than those with a history of allergy. There was no significant difference in those with a history of allergy, but the reason was unclear.

There was no association between an increase in NO_2_ concentration and pulmonary function. In power plant workers exposed to NO_2_, pulmonary functions were reduced significantly [38]. Gauderman et al. indicated that the odds ratio of asthma was 1.83 (95% CI: 1.04–3.22) when the outdoor concentration of NO_2_ increased by 5.7 ppb (IQR) [39]. Furthermore, Liu et al. conducted a study among children with asthma, and reported that an increase in NO_2_ concentration significantly reduced pulmonary function [40]. The concentration of NO_2_ measured in our study was relatively low; therefore, there may have been no influence on the respiratory system.

We had conducted the survey in the same area in the autumn of 2013, and found negative associations between pulmonary function and the concentrations of PM_2.5_, NO_2_, and BC among subjects with a history of asthma [41]. In the present study, to estimate the exposure to air pollutants accurately, we conducted measurements of the indoor concentrations of air pollutants in addition to outdoor concentrations. The subjects in the present study were different from those in the previous one, and this survey was conducted during spring. In the results of this study, an inverse association between pulmonary function and the concentrations of air pollutants among subjects with a history of asthma or allergy, which was compatible with the previous study. Therefore, subjects with a history of asthma or allergy were considered to be more susceptible to air pollutants. These adverse effects of air pollution may be modified by medication for asthma or allergy. However, in this study, information on the medication taken by the subjects was not obtained. The interaction with medication should be further evaluated.

This study has several limitations. One is that the number of subjects was small, resulting in an insufficient detection power; therefore, the influence of air pollution may have been underestimated. However, pulmonary function tests were conducted every morning, and a significant reduction in pulmonary function related to increases in BC or O_3_ concentrations was observed, even though the number of subjects was relatively small. The second limitation is that the study period was short and was limited to one season. It has been reported that the concentrations and sources of air pollutants have seasonal variations. In the future, a large-scale survey involving a larger number of subjects should be conducted for a longer period. The third limitation is that only PEF and FEV_1_ were used as pulmonary function parameters. Although inflammation of the airway may be involved in a reduction of pulmonary function, it was not tested in this study. In the future, the inflammatory state of the airway should be evaluated objectively in addition to pulmonary function tests.

## Conclusion

In an isolated island without major artificial sources of air pollutants, changes in the concentrations of air pollutants acutely influenced the pulmonary function of healthy subjects. The results suggest that gaseous substances, such as O_3_, are sometimes generated indoors, and that the indoor concentration of O_3_ more markedly influences pulmonary function compared with its outdoor concentration. In subjects with a history of allergy, pulmonary function reduced with an increase in the concentration of PM_2.5_.

## Additional file

Additional file 1: Table S1. Associations between air pollutants and pulmonary function during the study period in each single- and two-pollutant model. (DOCX 23 kb)

## Abbreviations

BC: Black carbon
FEV_1_: Forced expiratory volume in 1 s
IQR: Interquartile range
NO_2_: Nitrogen dioxide
O_3_: Ozone
PEF: Peak expiratory flow
PM_10-2.5_: Particulate matter between 2.5 and 10 μm in diameter
PM_2.5_: Particulate matter ≤ 2.5 μm in diameter
