# Supplementary material for: Acute effects of air pollutants on pulmonary function among students: a panel study in an isolated island
Source: Environ Health Prev Med. 2017 Apr 4;22:33. doi: 10.1186/s12199-017-0646-3 (PMC5664589; doi:10.1186/s12199-017-0646-3)
Supplement: Additional file 1: Table S1. — Associations between air pollutants and pulmonary function during the study period in each single- and two-pollutant model. (DOCX 23 kb) [file 12199_2017_646_MOESM1_ESM.docx]

Table S1 Associations between air pollutants and pulmonary function during the study period in each single- and two-pollutant model.

|  |  | PEF (L/min) | | |  | FEV_1_ (mL) | | |  |
| --- | --- | --- | --- | --- | --- | --- | --- | --- | --- |
|  |  | Change | 95% CI | P Value |  | Change | 95% CI | P Value |  |
| Outdoor air pollutants | | |  | | |  |  |  | |
| PM_2.5_ | |  |  |  |  |  |  |  |  |
|  | Single | -4.57 | (-10.67, 1.52) | 0.141 |  | -41.19 | (-84.38, 1.99) | 0.062 |  |
|  | +O_3_ | -4.34 | (-10.60, 1.92) | 0.174 |  | -39.54 | (-84.01, 4.94) | 0.081 |  |
|  | +NO_2_ | -7.62 | (-14.65, -0.60) | 0.033 |  | -35.07 | (-84.68, 14.55) | 0.166 |  |
| PM_10-2.5_ | |  |  |  |  |  |  |  |  |
|  | Single | -2.71 | (-9.04, 3.62) | 0.401 |  | -37.50 | (-81.80, 6.79) | 0.097 |  |
|  | +O_3_ | -2.22 | (-9.13, 4.68) | 0.528 |  | -36.70 | (-85.36, 11.95) | 0.139 |  |
|  | +NO_2_ | -4.04 | (-10.81, 2.72) | 0.241 |  | -30.61 | (-77.90, 16.68) | 0.204 |  |
| BC | |  |  |  |  |  |  |  |  |
|  | Single | -3.53 | (-7.31, 0.25) | 0.067 |  | -27.28 | (-54.10, -0.46) | 0.046 |  |
|  | +O_3_ | -3.59 | (-7.37, 0.20) | 0.063 |  | -27.94 | (-54.81, -1.08) | 0.041 |  |
|  | +NO_2_ | -6.99 | (-11.74, -2.24) | 0.004 |  | -25.81 | (-60.00, 8.38) | 0.139 |  |
| O_3_ | |  |  |  |  |  |  |  |  |
|  | Single | -3.84 | (-15.42, 7.74) | 0.515 |  | -30.93 | (-112.84, 50.99) | 0.459 |  |
|  | +PM_2.5_ | -2.00 | (-13.88, 9.89) | 0.742 |  | -13.83 | (-98.19, 70.54) | 0.748 |  |
|  | +PM_10-2.5_ | -2.20 | (-14.83, 10.43) | 0.732 |  | -3.81 | (-93.91, 86.29) | 0.934 |  |
|  | +BC | -4.32 | (-15.91, 7.26) | 0.464 |  | -35.82 | (-117.76, 46.13) | 0.391 |  |
| NO_2_ | |  |  |  |  |  |  |  |  |
|  | Single | 2.18 | (-3.67, 8.02) | 0.466 |  | -28.23 | (-69.40, 12.95) | 0.179 |  |
|  | +PM_2.5_ | 5.83 | (-0.90, 12.57) | 0.089 |  | -12.04 | (-59.29, 35.22) | 0.617 |  |
|  | +PM_10-2.5_ | 3.50 | (-2.75, 9.75) | 0.272 |  | -18.75 | (-62.68, 25.19) | 0.402 |  |
|  | +BC | 8.75 | (1.42, 16.08) | 0.019 |  | -3.65 | (-56.04, 48.75) | 0.891 |  |
| Indoor air pollutants | | | | | | | |  |  |
| PM_2.5_ | |  |  |  |  |  |  |  |  |
|  | Single | -3.21 | (-8.63, 2.20) | 0.245 |  | -9.58 | (-48.50, 29.34) | 0.629 |  |
|  | +O_3_ | -2.08 | (-7.52, 3.36) | 0.453 |  | -6.97 | (-46.11, 32.17) | 0.727 |  |
|  | +NO_2_ | -4.71 | (-10.85, 1.44) | 0.133 |  | -5.37 | (-49.58, 38.84) | 0.812 |  |
| O_3_ | |  |  |  |  |  |  |  |  |
|  | Single | -8.03 | (-13.02, -3.03) | 0.002 |  | -20.14 | (-55.36, 15.09) | 0.262 |  |
|  | +PM_2.5_ | -7.77 | (-12.81, -2.73) | 0.003 |  | -19.35 | (-54.83, 16.14) | 0.285 |  |
|  | +NO_2_ | -8.02 | (-13.02, -3.03) | 0.002 |  | -20.03 | (-55.29, 15.23) | 0.265 |  |
| NO_2_ | |  |  |  |  |  |  |  |  |
|  | Single | 0.81 | (-3.96, 5.59) | 0.738 |  | -9.88 | (-43.22, 23.46) | 0.561 |  |
|  | +PM_2.5_ | 2.78 | (-2.64, 8.19) | 0.315 |  | -7.74 | (-45.61, 30.13) | 0.688 |  |
|  | +O_3_ | 0.76 | (-3.99, 5.51) | 0.754 |  | -9.71 | (-43.01, 23.59) | 0.567 |  |

PEF, peak expiratory flow; FEV_1_, forced expiratory volume in 1 second; CI, confidence interval; PM_2.5_, particulate matter ≤ 2.5 μm in diameter; PM_10-2.5_, particulate matter between 2.5 and 10 μm in diameter; BC, black carbon; O_3_, ozone; NO_2_, nitrogen dioxide.

* Mean changes in PEF or FEV_1_ associated with an increase in the interquartile range of each air pollutant.
